# Transfer Learning in Cancer Genetics, Mutation Detection, Gene Expression Analysis, and Syndrome Recognition

**DOI:** 10.3390/cancers16112138

**Published:** 2024-06-04

**Authors:** Hamidreza Ashayeri, Navid Sobhi, Paweł Pławiak, Siamak Pedrammehr, Roohallah Alizadehsani, Ali Jafarizadeh

**Affiliations:** 1 Student Research Committee, Tabriz University of Medical Sciences, Tabriz 5165665811, Iran; hamidrezaashayeri79@gmail.com; 2 Nikookari Eye Center, Tabriz University of Medical Sciences, Tabriz 5165665811, Iran; navidsbg.ns1998@gmail.com (N.S.); ali.jafarizadeh.md@gmail.com (A.J.); 3 Department of Computer Science, Faculty of Computer Science and Telecommunications, Cracow University of Technology, Warszawska 24, 31-155 Krakow, Poland; 4 Institute of Theoretical and Applied Informatics, Polish Academy of Sciences, Bałtycka 5, 44-100 Gliwice, Poland; 5 Faculty of Design, Tabriz Islamic Art University, Tabriz 5164736931, Iran; s.pedrammehr@gmail.com; 6 Institute for Intelligent Systems Research and Innovation (IISRI), Deakin University, Burwood, VIC 3216, Australia; r.alizadehsani@deakin.edu.au; 7 Immunology Research Center, Tabriz University of Medical Sciences, Tabriz 5165665811, Iran

**Keywords:** artificial intelligence, cancer, deep learning, genetics, protein, transfer learning, gene mutation, syndrome

## Abstract

**Simple Summary:**

Transfer learning is a technique utilizing a pre-trained model’s knowledge in a new task. This helps reduce the sample size and time needed for training. These characteristics of transfer learning make it a perfect candidate to use in genetic research. The aim of our study is to review the current uses of transfer learning in genetic research. Here, we overview the use of transfer learning in the mutation detection of different cancers (lung, gastrointestinal, breast, glioma), gene expression, genetic syndrome detection (Down’s syndrome, Noonan syndrome, Williams–Beuren syndrome) based on the phenotype of patients, and identifying possible genotype–phenotype association. Using transfer learning in model development increases the final performance of the model compared with models trained from scratch.

**Abstract:**

Artificial intelligence (AI), encompassing machine learning (ML) and deep learning (DL), has revolutionized medical research, facilitating advancements in drug discovery and cancer diagnosis. ML identifies patterns in data, while DL employs neural networks for intricate processing. Predictive modeling challenges, such as data labeling, are addressed by transfer learning (TL), leveraging pre-existing models for faster training. TL shows potential in genetic research, improving tasks like gene expression analysis, mutation detection, genetic syndrome recognition, and genotype–phenotype association. This review explores the role of TL in overcoming challenges in mutation detection, genetic syndrome detection, gene expression, or phenotype–genotype association. TL has shown effectiveness in various aspects of genetic research. TL enhances the accuracy and efficiency of mutation detection, aiding in the identification of genetic abnormalities. TL can improve the diagnostic accuracy of syndrome-related genetic patterns. Moreover, TL plays a crucial role in gene expression analysis in order to accurately predict gene expression levels and their interactions. Additionally, TL enhances phenotype–genotype association studies by leveraging pre-trained models. In conclusion, TL enhances AI efficiency by improving mutation prediction, gene expression analysis, and genetic syndrome detection. Future studies should focus on increasing domain similarities, expanding databases, and incorporating clinical data for better predictions.

## 1. Introduction

Artificial intelligence (AI) and its subtypes, namely machine learning (ML) and deep learning (DL), have pioneered a new vision in every subject of medicine. From aiding in drug discovery to enhancing cancer diagnosis, AI is becoming an inseparable part of the future. As a subtype of AI, ML leverages the provided data to train and identify a pattern to complete tasks [1,2]. DL is a subtype of ML that uses neural networks in which information moves from one layer to the other to find the best route for data processing [3,4]. The learning process of an AI is complex. However, it can be categorized into four main types: supervised, unsupervised, semi-supervised, and reinforcement learning [5,6]. If labeled data are used to train the model, it is called supervised, and if the raw data are used, it is called an unsupervised ML. Semi-supervised learning uses both labeled and raw data in the learning process [7]. The choice of learning methods is mainly based on the task we assign to AI models. Supervised and semi-supervised learning is often used for prediction tasks, and unsupervised learning is beneficial in descriptive tasks [8].

Developing AI models for predictive tasks, such as classification, presents unique challenges. For instance, an expert is required to label the data, which is time consuming. Additionally, processing labeled data by AI requires more time than unlabeled data. The need for an expert, the labeling process, and the data processing in the training phase are all significant challenges in AI model development for predictive tasks [9,10,11]. However, methods like transfer learning (TL) have been developed to address these challenges [11]. TL uses an ML model that has been pre-trained in one task (named the source domain), which is then related to the current task (called the target domain). TL reduces the training sample size, resulting in faster training [12]. Models that use TL are also reported to have a higher performance than models trained on the dataset for the first time [13]. TL can also be divided into three types based on the labeling condition data used during the source and target domains: transductive TL (which uses labeled data in the source domain and unlabeled data in the target domain), inductive TL (which uses labeled data in target domain), and unsupervised TL (which does not use labeled data at all) [14]. The tasks given to the AI (both source domain and target domain) affect the preferred method of using TL. When the tasks in both domains are the same, an inductive TL is usually selected, and if the tasks are different but relatable, the other two are chosen [14].

The human genome is made up of 46 chromosomes, and is estimated to contain fewer than 20,000 protein-coding genes [15], with this number accounting for only 2% of the total human genome [16]. Genes are responsible for cells’ function; to do this function, multiple components are involved. Each gene is made up of different sections, like a promotor sequence, various exons, and introns [17,18,19]. However, other compartments, like RNA polymerase, transcription factors (TF), and enhancer sequences, are essential for the expression of a gene [20,21]. Humans also have a diversity in their genomes called alleles, which affect how they respond to different diseases. Dysfunction in gene structure and function is a significant pathophysiology of human disease. Some of these are congenital (e.g., Noonan syndrome) and some are acquired later in life (e.g., UV-induced DNA damage) [22,23]. Detecting human genetic function is crucial as it can affect the treatment options of patients (e.g., estrogen receptor (ER) mutation in breast cancer [24]). Nevertheless, these complexities make human genetic research (e.g., mutation detection, gene expression, and different gene alleles) a challenging, expensive, and time-consuming process [25]. TL’s benefits, as discussed, can offer a practical solution for these challenges in genetic research. We aim to overview some of the uses of TL in human genetic research, such as in gene expression, mutation, genetic syndrome detection, and genotype–phenotype association.

## 2. Literature Search Strategy

A comprehensive online search was conducted on the PubMed, Scopus, and Google Scholar databases until April/2024 to find the relevant studies, with the following proposed keywords: “genomic sequencing”, “mutation”, “mutation identification”, “genotyping”, “genetic mutation information”, “genetic”, “cancer”, “oncogene”, “tumor-related gene” and “transfer learning.” Only high-quality original literature in English that used TL in mutation detection, genetic syndrome detection, gene expression, or phenotype–genotype association were included in this review. There was no restriction regarding time and country of origin.

## 3. Mutation Identification

Mutation is defined as a change in a DNA sequence. There are many types of genetic mutations affecting genes and chromosomes [26,27]. These mutations can affect the gene expression or the protein function and structure [28] and are a cornerstone for many diseases and genetic diseases (e.g., thalassemia) [29,30,31]. Phospholamban is a protein in cardiac myocytes that interacts with Ca^2+^ pumps [32]. Mutations in the Phospholamban gene have been found to cause arrhythmias, cardiomyopathies, and sudden cardiac death [33,34]. Lopes et al. (2021) [35] targeted the identification of p.Arg14del as a mutation from patients’ electrocardiography (ECG). Convolutional neural network (CNN) was first trained to differentiate the sexes of the patients based on their ECG (source domain). Then, TL was applied to tune the model for mutation identification (target domain). This approach resulted in an area under the receiver operator curve (AUROC) of 0.87 with 80% sensitivity and 78% specificity.

Mutations in genes regulating cell growth or cell death are a common and crucial pathophysiological change seen in cancers. Mutations cause cancer and can affect the disease course, progression, patient’s survival, and treatment options [29,36]. Thus, identifying these mutations in cancer patients is of various clinical importance. TL can be handy in identifying these mutations in lung, gastrointestinal, brain, and breast cancers [10]. Table 1 summarizes the uses of TL in the mutation detection of different diseases.

### 3.1. Lung Cancer

Lung cancer is the leading cause of cancer death worldwide [53]. It can be divided into four main categories: lung adenocarcinoma, large cell carcinoma, squamous cell carcinoma (SCC), and small cell carcinoma [54]. EGFR mutation is the most common oncogenic change found in non-small cell lung cancers (NSLC) (including adenocarcinoma, squamous cell carcinoma, and large cell carcinoma) [55], and anti-EGFR therapy is used in the treatment of NSLC [56]. Xiong et al. (2019) [37] applied a ResNet-101 model to identify EGFR mutation status based on the chest computed tomography (CT) of 1010 patients with lung adenocarcinoma. They had two 2D CNN models, one pre-trained on the ImageNet dataset (source domain) and the other trained from scratch. TL-applied 2D-CNN models outperformed the 2D-CNN model trained solely on the CT images. The 2D-CNN model that was fine-tuned on the transverse plane had an AUROC of 0.766, and the 2D-CNN model that was fine-tuned on multi-view plane CT images had an AUROC of 0.838. Meanwhile, the trained 2D-CNN models had a lower AUROC than the fine-tuned models (AUROC for transverse plane input was 0.712 and for multi-view plane was 0.733). These data show a high performance for TL models. A CNN model was trained from scratch using 3D volume images as input, achieving an AUROC of 0.809. When comparing 3D-CNN and 2D-CNN models trained from scratch, it is evident that 3D images can improve performance. However, there is a risk of overfitting with 3D images. Therefore, utilizing the power of TL is recommended to mitigate this risk [37].

Similar to the previous study, Shao et al. (2024) [51] used a pre-trained CNN model to identify the EGFR mutation in lung adenocarcinoma. They used patients’ positron emission tomography (PET)/CT images as input data for a pre-trained 3D CNN model. The best performance was achieved when they used PET/CT images alongside the clinical data to make a diagnosis (AUROC: 0.73). They also trained two models from scratch: one with CT images as input and the other with PET images. AUROC of the models trained from scratch was 0.544 (input data was CT) and 0.573 (input data was PET). Compared with models with similar input data but that had been pre-trained (AUROC of 0.701 when using CT images as input and 0.645 when using PET images), the former approaches had a lower performance [51].

Silva et al. (2021) [43] used a TL to apply an unsupervised trained conventional autoencoder on CT images of patients with lung cancer. Their source domain used image segmentation and lung nodule detection based on CT images of the LIDC-IDRI dataset. They used three different input data sets for EGFR mutation status in patients. The best AUROC was achieved when only one lung was used to provide input data (0.68) [43]. Hiam et al. (2022) [44] used a pre-trained ResNet-50 model to identify EGFR mutation based on the magnetic resonance imaging (MRI) of patients with NSLC and brain metastasis. They achieved an accuracy of 89.8% with a sensitivity of 68.7% and a specificity equal to 97.7% [44].

Tumor mutation burden is proposed as a new marker for immunotherapy in NSLC [57]. In 2023, Dammak et al. [49] tried to identify a high tumor mutation burden in lung SCC using histopathologic images. Their models achieved an AUC of 0.6–0.8 [49]. Sometimes there is diversity between the cancer cells in a single tumor, called tumor heterogeneity [58]. This heterogeneity can affect the therapeutic response of patients because different colonies of the tumor have different properties [59]. To identify this diversity, Zheng et al. (2022) [47] used a specific TL method, called transfer component analysis (TCA). Using TCA, they tried to overcome the differences between the source and target domains. They tested their model in clonal populations with different proportions (5%, 10%, 15%, 20%, 25%, 30%) and achieved 81.18–92.1% accuracy for each proportion. They also tested the model on actual human data from the WES dataset, with 93.6–97.45% accuracy [47].

### 3.2. Breast Cancer

Some important mutations in breast cancers are ER, progesterone receptor (PR), and human epithelial growth factor receptor 1/2 (HER1/2) mutations, which can affect the behavior of cancer and its treatment options [60,61,62]. Furtney et al. (2023) [50] tried to determine breast cancer molecular categorization by using MRI. Their feature extractor model was pre-trained on the ImageNet dataset and achieved an AUROC of 0.871 in TCGA and 0.895 on the I-SPY2 dataset [50]. A survey by Rashid et al. (2024) [52] tried to identify HER2 mutation from histopathologic images of breast cancers. They used two databases (HER2SC and HER2GAN), a pre-trained ResNet-50 as a feature extractor, NSGA-II as a feature selector, and SVM for classification. They increased the method’s accuracy from 90.75% to 94.4% by increasing the number of features (from 549 to 633) and increasing the ratio of selected features (from 26.81% to 30.91%) [52].

### 3.3. Gastrointestinal Tract Cancer

Colorectal cancer is the third most common cancer around the world [63]. Microsatellite instability is a feature of cancers that represent DNA-mismatch repair system defects [64]. In the case of colorectal cancer, it is reported to improve the patient’s prognosis [65]. Cao et al. (2020) [38] trained their model on colorectal cancer histologic images from the TCGA-COAD database and used TL to generalize the model on the Asian-CRC database. A model trained on the TCGA-COAD performed an AUROC of 0.6497 in Asian-CRC, but, after applying the TL, the performance rose to an AUROC of 0.8504. By increasing the number of cases from Asian-CRC in the fine-tuning process, the performance increased to 0.9264 [38]. One of the problems with AI research is that the model’s performance may fall in an environment other than that of the study’s data (like the first time the model was tested on Asian-CRC). However, TL and fine-tuning of the model are methods by which to avoid over-fitting and decreased performance. Li et al. (2022) [45] targeted the STK11, TP53, LRP1B, NF1, FAT1, FAT4, KEAP1, EGFR, and KRAS mutation status detection for colorectal cancer based on histopathologic images and a pre-trained AI model on ImageNet [45].

Gastrointestinal stromal tumor (GIST) is cancer from Cajal cells in the gastrointestinal tract [66]. Thirty percent of GISTs are malignant and can occur anywhere throughout the gastrointestinal tract [67]. Two of the most mutated genes in GIST are the KIT and PDGFRA gene mutations [68]. Identifying these mutations is vital as there is specific therapy for them [69,70]. A CNN model was proposed by Liang et al. (2021) [42] to identify the KIT and PDGFRA gene mutations based on the histologic images. Pre-trained models on ImageNet were used to predict these drug-sensitive mutations. Their model achieved an accuracy of 70–85%. One of the features of AI in image processing is segmenting images into different parts, and one or all of the segmented parts can be used to learn and make a decision. DensNet-201 model achieved an accuracy of 81% (AUROC: 0.8832) when the decision was based on the nuclei picture and 79% (AUROC: 0.8562) when the cell without nuclei formed the input data [42].

TL was also applied to identify the tumor mutation burden of gastrointestinal cancer (gastric cancer and colon cancer) by Wang et al. (2020) [40]. They used eight pre-trained CNN models and histopathologic images to classify the tumor mutation burden of cancers into two groups: high mutation burden and low mutation burden. This method resulted in an AUROC between 0.68–0.82. They also reported their accuracy at the patch level (49–60%) instead of the patient level, reducing the accuracy numbers (reduction in VGG-19 was 19%, and in GoogleNet was 16%). This reduction is due to the possible heterogeneity of patients in the number of positive and negative patches [40].

### 3.4. Brain Cancers

**Figure 1:**
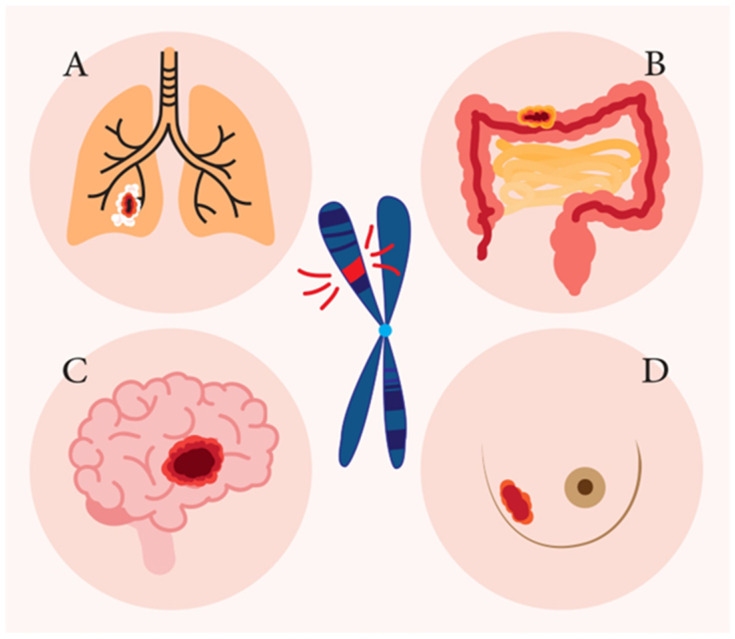
The role of mutations in different types of cancers. (**A**) Lung cancer, (**B**) gastrointestinal cancer, (**C**) brain cancer, and (**D**) breast cancer.

Glioma is a common primary tumor of the central nervous system [71] originating from glial cells [72,73]. Isocitrate dehydrogenase (IDH) mutation is one of glioma’s most common and essential mutations [74]. Zeng et al. (2022) [46] attempted to identify the type of IDH mutation from a multi-model MRI of glioma patients. They utilized a pre-trained model on ImageNet for feature extraction, and the model’s overall performance in IDH status prediction resulted in an AUROC of 0.86 with a sensitivity of 77.78% and specificity of 75% [46]. Figure 1 illustrates the role of mutations in common types of cancers.

### 3.5. Other Cancers

Models proposed until now have specialized in a specific type of cancer. Nevertheless, Fu et al. (2020) [39] used a pre-trained feature extractor DL model on histopathologic images of 28 different cancers (e.g., thyroid tumors, uterine cancer, glioma, breast cancer, etc.) and identified mutations (RB1, PTEN, CSMD1, PPP2R2A, BRAF, TP53, EGFR mutations). Their model achieved an average AUROC of 0.98 [39].

## 4. Gene Expression

Gene expression is an important, vast, and complicated part of human physiology. Various DNA sequences (e.g., promoters, enhancers, silencers) and proteins (e.g., TF, RNA polymerase, etc.) are involved in gene expression [26,27], and studying their interaction is complex. The application of TL has been shown to be useful in promotor–enhancer interactions, DNA methylation sites, TF-DNA interactions, and the effect of nucleotide polymorphism on gene expression. Table 2 summarizes the results of the articles in this section.

### 4.1. DNA Sequences Related to Gene Expression

Zhaung et al. (2019) [75] used a pre-trained CNN model for feature extraction to predict enhancer–promotor interactions of six cell lines. They used two different TL approaches: (1) training on five cell lines, then using the TL to train and test the model on the sixth cell line, and (2) training the model on all six cell lines, then using TL to train and test a specific cell line. In the second approach, AUROC and the area under the precision-recall curve (AUPRC) were higher. Additionally, epochs used in the second approach’s second training were fewer than in the first approach (20 vs. 24). Notably, these methods outperformed the model that had been trained and tested on a specific cell line from the start with fewer epochs [75]. Zhang et al. (2021) [78] used the same TL approach and the same cell lines as Zhaung et al. (2019) [75]. They also trained a model from scratch, and, as they report, utilizing TL increased the F1-score by 0.66–0.69 and AUROC/AUPRC by >0.4 [78].

A similar survey by Jing et al. (2020) [77] trained the DL utilizing TL for enhancer–promotor interaction prediction. Two different training strategies were used: (1) training their model on one cell line and then transferring the experience to test on a particular cell line, and (2) training a DL on data from all seven cell lineages and testing on a particular cell. The second method outperformed the first method in all seven cells, possibly due to the increased training data size. They also found that the higher the number of cell lines used in the source domain, the higher the performance [77].

To identify DNA regulator elements and possible binding sites for TF, Salvatore et al. (2023) [82] pre-trained a DL model in order to identify representative DNase I hypersensitive sites in a specific cell type in order to predict the same regulatory sequences. Their model achieved an AUROC between 0.79–0.89, depending on the cell lineage [82]. Mehmood et al. (2024) [83] tried to differentiate the encoder DNA sequences from non-encoder DNA sequences. To achieve this goal, they first trained a language model AI to predict a group of nucleotides based on the previous nucleotides. This training process can be classified as unsupervised training and then applied to the pre-trained model in the enhancer identification process. They also used the AI to predict the strength of the enhancer. This method achieved an accuracy of 84.3% for encoder identification and 87.5% for encoder strength prediction [83].

### 4.2. DNA Methylation

DNA methylation is an epigenetic change affecting gene expression. Based on the methylation site, it can increase or decrease the expression of genes [92,93]. In mammals, cytosine is the most common nucleotide that goes through the methylation process and converts to 5-methylcytosine [94]. However, methylation of other nucleotides can also significantly impact gene expression and disease course. The O6-methylguanine DNA methyltransferase (MGMT) promotor methylation decreases the gene expression and improves glioma response to radiotherapy and alkylating agents [95,96]. Sakly et al. (2023) [80] used a pre-trained CNN model to predict the MGMT promotor methylation status based on the multimodal MRI images of glioma patients. They used the TL to transfer the convolutional layers of the CNN model and build a new classifier for this task. They used two models (ResNet-50 and DenseNet-201), reaching an accuracy of 100%, but the ResNet50 model had fewer layers and took less elapsed time [80].

NanoCon is a DL model that Yin et al. (2024) proposed in order to predict 5-methylcytosine [85]. They used the genetic data of Arabidopsis thaliana and Oryza sativa provided by NCBI and EnsemblePlants, with over 18,496,029 sites (10.83% of which were methylated). The NanoCon model was trained on the A. thaliana genome and used to identify the 5-methylcytosine sites on O. sativa (precision was between 90–100%). However, when the model was trained on the O. sativa and tested on the A. thaliana, the precision dropped to 40–50% [85]. This reduction may be due to the smaller genetic data of O. sativa compared with A. thaliana (8,060,024 vs. 10,436,005), to the unbalanced ratio of methylation sites between the two species’ databases (28% vs. 2%), or to the differences between the spices. They also trained the on-cytosine motifs and tried to predict methylation sites of the different cytosine motifs. They found that CpG and CHG motifs are the best motifs to train on. All of these emphasize that the training data should be chosen carefully in order to obtain the best results when using TL [85].

4-methylcytosine is another important epigenetic change in human DNA. Yao et al. (2024) [89] proposed DeepSF-4mC, an AI model to predict the 4-methylcytosine sites in DNA. They trained the model on DNA sequence data of three species: A. thaliana, Caenorhabditis elegans, and Drosophila melanogaster. Then, one hot TL method was applied to identify 4-methylcytosine in each species and achieved an accuracy of 86.1–90.7%, sensitivity of 88–92.5, and specificity of 84.2–88.8%. The performance of the DeepSF-4mC was lowest in A. thaliana and the highest in C. elegans and still outperformed similar studies that did not use TL [89].

**Figure 2:**
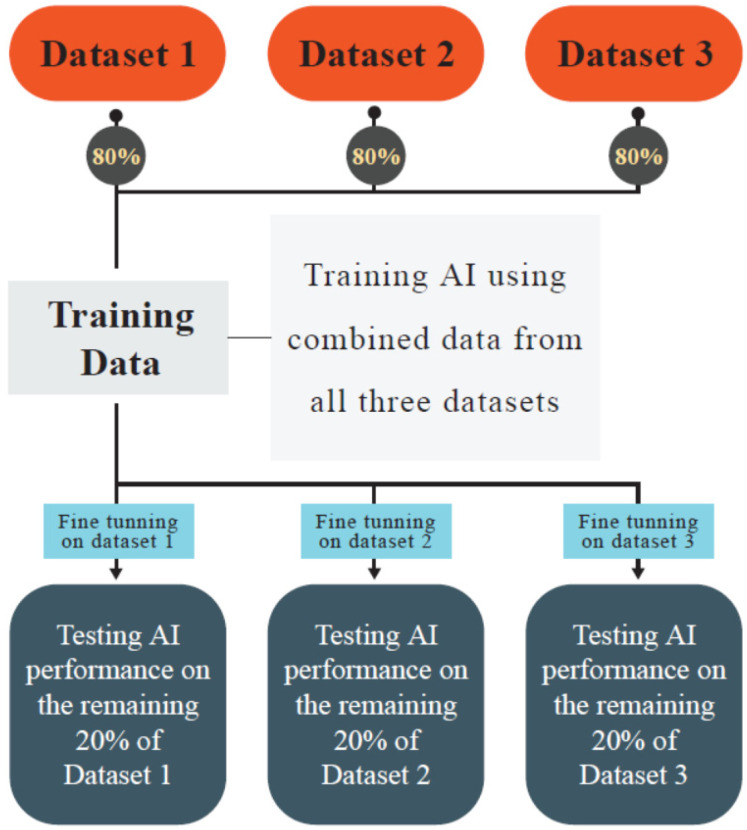
The figure illustrates the transfer learning process which involves gathering data from various datasets, fine-tuning the acquired data, and subsequently testing the datasets.

In a study by Li et al. (2023) [81], DNA sequences of 15 species were included and used to train a CNN-based model to predict 5-hydroxymethylcytosine, 4-methylcytosine, and 6-methyladenosine methylation sites. After training, they fine-tuned the model to identify the desired methyl nucleotide in a particular species. The framework of this method is similar to that of the previous studies by Zhuang et al. (2019), Jing et al. (2021), and Yao et al. (2024) [75,77,78,89]: training a model on all of the different data and fine-tuning for each particular (Figure 2 presents the abstract of this method). The advantage of using this method is that the source and target domains are relatively similar. This way, Li et al. (2023) [81] could, on average, increase their AUROC and accuracy. EpiTEAmDNA’s accuracy in all datasets was above 75%, and their performance in predicting methyl nucleotides of humans had an accuracy of >90% [81].

### 4.3. Other Elements Involved in Gene Expression

TL was utilized to predict the TF and DNA motif binding by Kalakoti et al. (2023) [79]. Their method to investigate the binding of TF and DNA sequences was K-mer-based and included 26 different TF. With this model, they achieved an accuracy of 95.6% [79]. Histopathologic images of cancer are also a good source for gene expression and predicting their response to the therapy. Li et al. (2022) [45] trained and fine-tuned a pre-trained CNN model in order to identify their immune-related gene expression in colorectal cancer images obtained from TCGA. Their targeted genes included PD-L1, CD3G, and TNFRSF9 [45].

Nucleotide polymorphism can directly cause a change in gene expression. Some single nucleotide polymorphisms (SNP) can have such powers and can change in genes [97]. These SNP affect the genes by affecting the expression of quantitative trait locus (eQTL) [98]. If these eQTLs act on a nearby gene, they are called cis-eQTL [99]. In 2024, Zhang et al. [91] used a pre-trained TLegene model on the GTEx database to identify cis-SNPs in the TCGA database. To make the training and testing data more similar, the cancers included in the TCGA were the same as those in the GTEx database and included ten different cancers (e.g., adrenocortical, breast, lung SCC, colon, ovarian, etc.). By utilizing this method, they could discover 81 genes shared between the former cancers and 88 genes only for one of the cancers [91]. Curtailing the possible genes can happen faster and more efficiently using TL identification.

## 5. Genetic Syndromes

Down’s syndrome (DS) is a common chromosomal mutation found in 1:1000 live births [100]. Three types of abnormalities seen in chromosome 21 can cause DS: free trisomy 21, mosaic trisomy 21, and Robertsonian translocation trisomy 21 [101]. Karyotyping is the gold standard method used to diagnose DS, but it is rather time consuming. Wang et al. (2023) [102] applied TL to image segmentation of human chromosomes in the metaphase stage and classify them. Their database contained data from ADIR (*n* = 180), BiolmLab (*n* = 119), and their private database (*n* = 1084). They compared their models (Swin Transformer) to AI models trained from scratch (ResNet-50 and SE-ResNeXt-50). Swin Transformer achieved an accuracy/precision of 96.47%/90.91% in DS detection compared with 95.29%/86.96% for ResNet-50 and 91.76%/76.92% for SE-ResNeXt-50 [102]. These results imply the role of TL in increasing the performance of DL models.

Although genetic testing is the best way to diagnose DS, it is not usually used in everyday practice. Karyotyping starts with a high clinical suspicion of the chance of a DS in clinicians based on an individual’s features and phenotypes. AI can assess these features inexpensively, serving as a screening method by which to identify potentially high-risk patients for referral to karyotyping. VNL-Net is a TL-based feature extractor proposed by Raza et al. (2024) [103] to differentiate healthy children from DS by their facial images. This method achieved an accuracy/precision of 99%/99%, outperforming similar studies with accuracy and performance of 85%/90% [103].

Noonan syndrome is a genetic disease caused by RAS/MAPK pathway mutation. Because it is a rare disease, there are no screening tools for diagnosis at birth, and a clinician will suspect it based on the phenotype of patients. TL was used to train a DL model to differentiate children with Noonan syndrome from children without Noonan syndrome based on their facial images. A total of 420 children (127 patients with Noonan syndrome, 163 healthy controls, and 130 patients with other dysmorphic syndromes) were included. Patients were from three different age groups (infant, childhood, adolescence) and had different mutations (e.g., PTPN11, BRAF, RAF1, …). DL’s best results were an AUROC of 0.9797 ± 0.0055 and an accuracy of 92.01% ± 1.38% in distinguishing Noonan syndrome from healthy controls. They also tested the DL model to identify Noonan syndrome from patients with other genetic syndromes, and DL still outperformed an expert human geneticist (accuracy 81% vs. 61%) [104].

Williams–Beuren syndrome (WBS) is also a genetic disorder but is rarer than Noonan syndrome (1 in every 7500 vs. 1 in 1000–2500) [105,106]. Diagnosis is made when clinicians suspect WBS from phenotype and ask for genetic tests. TL was used to avoid overfitting DL models. One hundred four WBS and 236 control (145 healthy and 91 cases with other genetic syndromes) photographs were enrolled in this study. The best-achieved accuracy was 92.7% ± 1.3% and AUROC of 0.896 ± 0.013. All of the DL models in this study performed better than expert human operators in diagnosing WBS (worst DL model accuracy 85.6% vs. best human accuracy 82.1%) [107].

Another study tried to use 13 genetic syndromes, including WBS, Noonan syndrome, and DS facial images, as input for a previously trained VGG-16 model on the face. Four hundred fifty-six photographs were involved in this study (228 patients, 228 controls), and the model achieved an accuracy of 88.6% ± 2.11% and an AUROC of 0.9443 ± 0.0276 [108]. Comparing these results with the best accuracy achieved by five professional pediatricians (79.83%) shows DL’s superiority in detecting genetic disorders based on photographs.

An innovative study by Artoni et al. (2019) [109] tried to utilize an animal model DL to identify Rett syndrome patients. Their ConvNetAch model was trained to identify mice with autism spectrum disorder (ASD) via pupil fluctuation. Pupil fluctuation in ASD patients results from their cholinergic impairment [110]. Because both ASD and Rett syndrome patients have some degree of cholinergic system dysfunction, they used the TL to detect Rett syndrome. The only difference was that the input DL data for cholinergic activity in Rett syndrome patients was heart rate variation data. This study included 75 girls (35 with Rett syndrome, 40 typically developing). By using this approach, they reduced the size of the training sample (*n* = 20) and increased the accuracy (accuracy when using TL: 82%, accuracy when not using TL: 72%). They also reported an increase in the performance of TL when the training data was larger (*n* = 40, accuracy: 87%) [109].

## 6. Genotype–Phenotype Association

All of the studies in the last section used the phenotype of a patient to predict their genetic syndrome. However, TL can assist with other tasks related to genotype–phenotype association, like linking the effects of different mutations on the structure of proteins. A survey by Petegrosso et al. (2017) [111] took a unique approach to identifying phenotype–genotype association. They utilized AI power to identify new gene functions in relation to a phenotype. The Human Protein Reference Database (HPRD) was used to build a protein–protein interaction (PPI) network. Then, they trained their model to identify genes associated with a specific phenotype on the PPI network and the Human Phenotype Ontology (HPO) project. They used a TL model to train their model to find the relationship between genetic ontology (GO) term–gene association (based on the PPI network) and HPO–gene association and combine these two to see the relation between GO–HPO data. The best AUROC achieved by their model for predicting genes associated with a phenotype was 0.778 [111].

Predicting protein structure and function is important, especially in terms of the way that drugs interact with these proteins. Cytochrome P450 (CYP) is a critical superfamily of enzymes involved in drug metabolism [112]. Their metabolic capabilities are different in individuals in the populations, and these changes cause different pharmacokinetic properties that are special to each individual. It is believed that these polymorphisms result from genetic variations of these enzymes [113]. Thus, Mclnnes et al. (2020) [114] tried to predict the function of CYP2D6 from genetic data. This method could provide crucial information in the emerging field of personalized medicine. They included 127 alleles in this study (31 for training, 25 for validation, and 71 for testing). They did not include the alleles that cause increased function, as these are caused by genetic duplication. They trained a CNN model named Hubble.2D6 to identify no-function and normal-function alleles of CYP2D6 and applied TL to identify the lack of function, decreased function, or normal function of CYP2D6 haplotypes. Hubble.2D6 achieved an accuracy of 88% in the validation set. Their test set contained alleles whose functions are as yet unknown, and AI predicted that 30 would have normal function, 36 would have decreased function, and 5 would have no functions [114].

Alderfer et al. (2022) [115] tried to differentiate different oncogenic retinal pigmental epithelium cells from normal ones and classify them as different mutation groups from the actin structure of cells. They used TL applied on a pre-trained CNN model on the ImageNet dataset. The model’s accuracy in distinguishing normal from oncogenic cells was 95–97% (based on cell culture), and in distinguishing different mutations was 81–88%. They also tested the ResNet-50 model in multiclass classification and achieved an accuracy of 80–82% [115]. Kirchler et al. (2022) [116] used a pre-trained DL on ImageNet and EyePACS to identify the genes associated with retinal images obtained from UB-biobank. Their method proposes 60 loci associated with the retina, 19 of which were common between models pre-trained on ImageNet and EyePACS database. Thirty-six of these 60 genes had been previously described to be associated with different retinal pathologies (e.g., myopia, diabetic retinopathy) [116].

In 2022, Zhang et al. [117] developed a supervised ML model to predict the function of proteins in different genetic missenses (source domain). Then, they used TL to predict the effects of various mutations in the function of calcium- and sodium-voltage-gated channels (target domain). The TL-based model was compared with a model trained on the basis of differentiating the dysfunction of voltage-gated channels based on their genetic code and achieved a higher AUROC (0.96 vs. 0.93). They also attempted to find the effect of mutations on the dysregulation of channels and categorize proteins as gain of function and loss of function. The AUROC of the TL model in this analysis (0.95) was higher than a model that had been trained from scratch [117]. Another similar study by Zheng et al. (2024) [118] leveraged a pre-trained model-trained 3D protein structure prediction from sequence and predicted the stability of proteins resulting from different mutations. They trained 27 different models, and none were found to predict the mutation that causes protein stabilization [118].

## 7. Limitations and Challenges

The included studies mostly used TL in two methods. Firstly, the use of a pre-trained model or trained a model on a source domain which is then tested on the desired target domain. The source domain in this method is not necessarily similar to the target domain and can be trained on datasets like ImageNet. Secondly, data from different domains (e.g., DNA data of different species) were mixed, and training data (source domain) were created. Then, the AI was fine-tuned on the remaining data (target domain) (e.g., AI was fine-tuned on DNA sequences of a particular species). This method was used by [75,77,78,81,89], and is demonstrated in Figure 2. Compared with the first method, the second method increases the similarities between source and target domains.

Despite advancements in TL, it still faces several limitations. These include domain dissimilarity, reliance on large pre-training datasets, low data quality, and a lack of explainable techniques. Moreover, domain mismatch is a significant issue. Additionally, the necessity for extensive pre-training data poses challenges, especially in specialized fields with limited datasets. Low-quality data and the “black-box” nature of this model further complicate its reliability and interpretability, hindering its effective application across various domains [14]. Using the incorrect source domain to train the model can reduce its performance in the target domain, a phenomenon known as negative transfer [119]. TL is a proposed method to reduce overfitting. However, an inappropriate source domain or the addition of too many parameters can also reduce the generalizability and cause overfitting of the TL model [12]. These disadvantages are important to notice, especially when there are no relevant source domains for the task.

ImageNet is a popular source domain for DL models that aim to act on image processing. This is a database of various images organized to help AI in visual object recognition tasks [120,121,122]. As discussed in this literature, using ImageNet as a source domain and applying TL to function the model on a target domain increases the performance. However, medical images have some unique features (e.g., medical images tend to have more noise than photographic images). These differences may increase the bias or decrease the performance from the optimal performance [123]. Increasing the similarity between the source and target databases to avoid these problems seems like an obvious choice. A possible solution that Zhang et al. (2023) have proposed is to detect only local similarities between two domains and apply TL to them [124,125].

Studies can also act on databases with similar data, like TCGA-COAD and Asian CRC, in the work by Cao et al. [38]. However, while using two databases, the differences between them (e.g., the age of subjects, details regarding obtaining the samples, etc.) should also be considered. It is also essential to select the source domain and target domain carefully. As the Yin et al. [85] survey reports, reversing the places of source and target domains results in a significant decrease in precision (from 90–100% to 40–50%).

## 8. Future Perspectives

TL will offer promising advancements in drug discovery by improving the identification of therapeutic targets and by predicting patient responses to treatments. Studies investigating gene expression and mutation detection provide an important source for possible therapeutic targets and help with drug discoveries. For example, Song et al. (2023) [126] have developed a model by which to predict cancer driver mutations. Their model reached an accuracy of >93% in cancer driver mutation identifications and even proposed a missense mutation in the RRAS2 gene as a possible candidate for such mutations. TL is also a useful tool for predicting patients’ responses to chemotherapy based on mutations and gene expressions. Chen et al. (2022) [127] used the RNA sequencing data for this task. Their model successfully predicted Cisplatin resistance in 85% of cells, but as they have mentioned, their prediction accuracy varies from cell line to cell line. These studies show a promising place for AI, and especially TL, techniques in drug discovery and personalized medicine.

The included studies also tried to identify genetic syndromes from their facial images. Due to their rare nature, detection is challenging, and AI can provide an accurate, fast, and cheap screening tool. Three of the included studies targeted only one syndrome but, most of the time, features in patients’ phenotypes guide clinicians to a specific diagnosis. However, these features are sometimes shared between two syndromes. For example, low-set ears are common in Down’s syndrome and Noonan syndrome [128,129]. To reduce the possible bias in future research, providing AI with epidemiologic data of an area and patients’ clinical data (age, sex, abnormalities in internal organs), along with facial features can help to distinguish the characteristics of multiple genetic syndromes from persons without such syndromes.

TL will significantly enhance the diagnosis of diseases by utilizing genetic data patterns, such as those used in diagnosing cancers like leukemia. A study by Mallick et al. (2023) [130] used DL to identify the gene expression data and classify acute lymphocytic leukemia (ALL) and acute myelocytic leukemia (AML). This method reached an accuracy of 98.21%. Another study by Nazari et al. (2020) [131] used genetic data to differentiate between healthy and AML individuals. Their achieved accuracy with the DL model was 96.67%. These studies focus on the total genetic data for the diagnosis rather than targeting a single gene to identify. However, the genetic patterns of diseases may overlap with each other and decrease performance when the task is to identify multiple diseases. However, none of the studies used the TL model, and it is suggested that, by correctly applying the TL, models can distinguish between multiple diseases without a major decrease in performance.

## 9. Conclusions

AI can act as a diagnostic device that predicts genetic mutations or finds new genes related to a disease. TL increases the efficiency of AI research by reducing the overfitting and decreasing the number of samples needed for the training. This literature has discussed the previous AI task in regard to the use of TL methods and the way in which studies have applied TL in their work. TL increased the performance of mutation prediction based on the images, determined the gene expression and the involved components in the process, predicted the genetic syndromes based on the phenotypes, and provided helpful information about possible genes associated with a disease and the effects of a particular mutation on protein function and structure. Additionally, by accurately predicting gene expression and proposing new mutations, TL can increase our knowledge of cancers and affect cancer classifications and gradings. By selecting the right source and target domains, an AI algorithm is capable of leveraging experience and adapting it to a new situation. For future studies, we recommend increasing the similarities between the former domains. Increasing the number of domains (databases) and samples will surely increase the performance. Additionally, adding patients’ clinical data can increase the likelihood of making the correct prediction.

## Figures and Tables

**Table 1:** Summary of the application of TL in mutation detection.

Author (Year)	Goal	Used AI * Models	Databases	Cancer/Pathologic Condition	Input Data	TL	AUROC	Sensitivity, Specificity	Accuracy/Precision
Xiong et al. (2019) [37]	EGFR mutation status (mutated vs. wild-type)	ResNet-101	Patients from 2013–2017	Lung adenocarcinoma (*n* = 1010)	Non-contrast-enhanced CT images	CNN model was pre-trained on ImageNet and fine-tuned on the CT images	Two-dimensional slice images in a transverse plane: 0.766	-	-
							Two-dimensional slice images in multi-view plane: 0.838		
Cao et al. (2020) [38]	Microsatellite instability	EPLA	TCGA-COAD (*n* = 429), Asian-CRC (*n* = 785)	Colorectal Cancer	Histopathology images	The model was trained in TCGA-COAD and then generalized on Asian-CRC	0.9264	-	-
Fu et al. (2020) [39]	Tumor mutations	PC-CHiP (based on Inception-V4)	TCGA	Twenty-eight cancers	Histopathology images of cancer and normal tissue (*n* = 17,396)	A pre-trained model on 1536 histopathologic features was used as a mutation predictor.	BRAF in thyroid tumors: 0.92	-	-
							PTEN in uterine cancers: 0.82		
							TP53 in uterine cancer: 0.8		
							TP53 in low-grade glioma: 0.84		
							TP53 in breast invasive carcinoma: 0.82		
Wang et al. (2020) [40]	Tumor mutation burden	ResNet-18	Data was downloaded from https://doi.org/10.5281/zenodo.2530835. [41]	Gastrointestinal cancer (*n* = 545) was split into two cohorts named TMB-STAD (*n* = 280) and TMB-COAD-DX (*n* = 265)	Histopathology images	TL-based CNN models were used to classify the mutation burden	AUROC in TMB-STAD/TMB-COAD-DX ResNet-18: 0.73/0.77	-	Accuracy in patch level in TMB-STAD/TMB-COAD-DX: 0.52/0.57
		ResNet-50					ResNet-50: 0.71/0.76	-	Accuracy in patch level in TMB-STAD/TMB-COAD-DX: 0.53/0.6
		GoogleNet					GoogleNet: 0.75/0.78	-	Accuracy in patch level in TMB-STAD/TMB-COAD-DX: 0.55/0.59
		InceptionV3					InceptionV3: 0.74/0.73	-	Accuracy in patch level in TMB-STAD/TMB-COAD-DX: 0.52/0.57
		AlexNet					AlexNet: 0.68/0.76	-	Accuracy in patch level in TMB-STAD/TMB-COAD-DX: 0.53/0.58
		VGG-19					VGG-19: 0.71/0.82	-	Accuracy in patch level in TMB-STAD/TMB-COAD-DX: 0.53/0.58
		SqueezeNet,					SqueezeNet: 0.7/0.75	-	Accuracy in patch level in TMB-STAD/TMB-COAD-DX: 0.57/0.49
		DenseNet-201					DenseNet-201: 0.73/0.79	-	Accuracy in patch level in TMB-STAD/TMB-COAD-DX: 0.55/0.6
Liang et al. (2021) [42]	KIT/PDGFRA gene mutation (KIT exon mutations, PDGFRA mutations, and KIT/PDGFRA wild-type mutation)	AlexNet	Three laboratories	Gastrointestinal stromal tumors (*n* = 365)	Histopathology images (*n* = 5153)	All models were pre-trained on ImageNet	-	-	Accuracy: (1) AlexNet: 70%
		Inception-V3							(2) Inception V3: 77%
		ResNet-101,							(3) ResNet-101: 84%
		DenseNet-201							(4) DenseNet-201: 85%
Silva et al. (2021) [43]	EGFR mutation status	A conventional autoencoder	LIDC-IDRI (*n* = 875), NSCLC-Radiogenomics (*n* = 116)	Lung cancer	CT images	A conventional autoencoder was trained on LIDC-IDRI data to perform segmentation and then TL was used to identify EGFR mutation status in the NSCLC-radiogenomics dataset.	when lung nodule was used as an input: 0.51	-	-
							when one lung was used as an input: 0.68		
							when both lungs were used as input data: 0.60		
Lopes et al. (2021) [35]	Phospholamban p.Arg14del mutation carries identification	CNN	18–60 years old patients	Cardiomyopathy	Lead I, II, V1–V6 ECG	AI trained to identify sex from ECG (*n* = 256,278) was fine-tuned and used to identify mutations (*n* = 155).	0.87	80%, 78%	-
Hiam et al. (2022) [44]	EGFR mutation status (positive vs. negative)	ResNet-50	Patients from 2006–2019	Non-small cell lung cancer with brain metastasis (*n* = 59)	T1C MRI	A pre-trained AI model on ImageNet data was used.	0.91	68.7%, 97.7%	Accuracy of 89.8%
Li et al. (2022) [45]	STK11, TP53, LRP1B, NF1, FAT1, FAT4, KEAP1, EGFR, KRAS mutation status	Xception (CNN-based model)	NCT-CRC	Colorectal cancer	Histopathologic images (100,000 were used for fine-tuning and 7180 for testing)	A pre-trained AI model on ImageNet was used in this study for the FE task.	AUROC data was provided in a graph	-	-
Zeng et al. (2022) [46]	IDH genotyping (mutant vs. wild-type)	MDSA for tumor segmentation and VGG-19 for feature extraction	BraTS 2019, patients from the hospital (2012–2020)	Grade II–IV glioma	T1W, T2W, T1C, FLAIR MRI (335 cases from BraTS and 110 from hospital)	VGG-19 feature extractor was pre-trained on ImageNet.	0.86	77.78%, 75%	Accuracy of 76.39%
Zheng et al. (2022) [47]	Detection of sub-clonal mutations	TLsub	Hg19 was used to create their own simulated data, data provided by Ma et al. [48], the WES dataset	DNA mutation, lung cancer, breast cancer	DNA sequence (these data were split into source and target domains)	TL was used to reduce the false positive rate by transferring the knowledge from the source domain to the target domain on simulated data	-	-	Accuracy of 81.18–92.1%, Precision of 86.92–91.8%
						they also tested their model on real human data	-	-	Accuracy of 73–90%, Precision of 67.69–90%
						they also tested their model on real human data from the WES dataset	-	-	Accuracy of 93.6–97.45%, Precision of 77.29–95.1%
Dammak et al. (2023) [49]	Predicting mutation burden of tumors	VGG-16	TCGA-LUSC	Lung SCC (*n* = 50)	Histopathology images (*n* = 50)	A pre-trained AI model on ImageNet data was used.	VGG-16: 0.8	-	-
		Xception					Xception: 0.7		
		NASNet-Large					NASNet-Large: 0.6		
Furtney et al. (2023) [50]	Molecular subtypes of breast cancer	RGCN (based on CNN)	TCGA-BRCA (*n* = 1040), I-SPY2 (*n* = 987), American Association For Cancer Research Project GENIE	Breast cancer	Dynamic contrast-enhanced MRI, pathologic testing results, radiologist reports, clinical attribution	EfficientNet-B0 CNN was pre-trained on the ImageNet dataset and used as a feature extractor.	On the TCGA dataset: 0.871 On I-SPY2: 0.895	-	-
Shao et al. (2024) [51]	EGFR mutation status	3D CNN	Patients from 2018–2022	Lung adenocarcinoma (*n* = 516)	PET/CT images and pre-clinical data	A pre-trained AI model called Model Genesis was acquired from GitHub	CT images: 0.701	74.6%, 58.5%	Accuracy: 68.8%
							PET images:0.645	54.9%, 65.9%	58.9%
							PET/CT images: 0.722	67.6%, 63.4%	66.1%
							PET/CT with clinical data: 0.73	67.6%, 65.9%	67%
Rashid et al. (2024) [52]	HER-2 mutation	ResNet-50 as the feature extractor, NSGA-II as the feature selector, and SVM as a classifier.	HER2SC and HER2GAN datasets	Breast cancer	Histopathology images	They used a pre-trained ResNet-50	-	on the HER2GAN dataset: 90.19%, 91.18%	90.8%, 90.31%
								on the HER2SC dataset with 633 features extracted: 93.73%, 98.07%	94.4%, 93.81%
								on the HER2SC dataset with 549 features extracted: 89.98%, 96.98,	90.75%/89.96%

* AI: artificial intelligence, AUROC: area under the receiver operator curve, CNN: convolutional neural network, CT: computed tomography, ECG: electrocardiography, FLAIR: fluid attenuated inversion recovery, MRI: magnetic resonance imaging, PET: positron emission tomography, SCC: squamous cell carcinoma, SVM: support vector machine, T1C: T1-contrast-enhanced, T1W: T1-weighted, T2W: T-2 weighted, TL: transfer learning.

**Table 2:** Summary of the applications of TL in gene expression research.

Author (Year)	Goal	Used AI Models	Databases	TL	Input Data	AUROC *	AUPRC *	F1-Score/MCC *	Accuracy/Precision *
Zhuang et al. (2019) [75]	Prediction of enhancer–promotor interactions	CNN (for FE and prediction)	Enhancer and promotor data provided by Singh et al. [76]	(1) FE task was trained on the data of five cell lines and was then paired with a specific fully connected layer to predict promotor–enhancer interaction in a specific cell line. (2) FE task was trained on the data of all cell lines and was then paired with a specific fully connected layer to predict promotor–enhancer interaction in a specific cell line	Six cell lines GM12878	(1) 0.96, (2) 0.98	(1) 0.88, (2) 0.92	-	-
					HeLa-S3	(1) 0.96, (2) 0.98	(1) 0.9, (2) 0.95	-	-
					HUVEC	(1) 0.97, (2) 0.99	(1) 0.9, (2) 0.95	-	-
					IMR90	(1) 0.96, (2) 0.98	(1) 0.9, (2) 0.92	-	-
					K562	(1) 0.96, (2) 0.99	(1) 0.9, (2) 0.95	-	-
					NHEK	(1) 0.97 (2) 0.99	(1) 0.92, (2) 0.96	-	-
Jing et al. (2020) [77]	Prediction of enhancer–promotor interactions	SEPT	Hi-C data, hg19	(1) AI was trained on one cell line and then tested on the second cell line (AI can be trained on each of six cell lines and then get tested on a particular cell line, and results are reported in a range). (2) AI was trained in half of the data from each cell line and then fine-tuned in a particular cell line.	DNA sequences of seven cell lines HeLa-S3	(1) 0.59–0.67 (2) 0.77	-	-	-
					GM12878	(1) 0.58–0.64 (2) 0.72	-	-	-
					K562	(1) 0.56–0.63 (2) 0.73	-	-	-
					IMR90	(1) 0.62–0.67 (2) 0.78	-	-	-
					NHEK	(1) 0.59–0.66 (2) 0.76	-	-	-
					HMEC	(1) 0.61–0.65 (2) 0.76	-	-	-
					HUVEC	(1) 0.57–0.69 (2) 0.78	-	-	-
Zhang et al. (2021) [78]	Prediction of enhancer–promotor interactions	EPIsHilbert	TargetFinder, SPEID	(1) AI was trained in data from five cell lines and then trained and tested in a particular cell line. (2) AI was trained in data from all six cell lines and then fine-tuned in a particular cell line	DNA sequences of six cell lines GM12878	(1) -, (2) 0.959,	(1) 0.946, (2) 0.97	F1 score: (1) -, (2) 0.917	-
					HeLa-S3	(1) -, (2) 0.95,	(1) 0.97, (2) 0.963	(1) -, (2) 0.907	-
					HUVEC	(1) -, (2) 0.938	(1) 0.949, (2) 0.953	(1) -, (2) 0.906,	-
					IMR90	(1) -, (2) 0.941	(1) 0.944, (2) 0.957	(1) -, (2) 0.898,	-
					K562	(1) -, (2) 0.954,	(1) 0.981, (2) 0.946	(1) -, (2) 0.925	-
					NHEK	(1) -, (2) 0.972	(1) 0.982 (2) 0.966	(1) -, (2) 0.949	-
Li et al. (2022) [45]	FE and its correlation with 907 immune-related gene expressions	Xception (CNN-based model)	NCT-CRC	A pre-trained AI model on ImageNet was used in this study for the FE task. (R correlation data was presented in the graph)	Histopathologic images (100,000 were used for fine-tuning and 7180 for testing)	-	-	-	-
Kalakoti et al. (2023) [79]	Prediction of TF-DNA interactions	TFactorNN (RNN-based model)	ENCODE project, hg19, JASPAR, HOCOMOCO	A pre-trained DNABERT model creates 380-dimensional DNA sequences, and an Att-biLSTM model is used to pre-train and learn long DNA sequences.	ChIP-seq data, ATAC-seq data, TF-binding motifs, input data of three cell lines (Hela-S3, k562, and GM12878)	-	-	0.83/0.65	Accuracy of 95.6%
Sakly et al. (2023) [80]	Prediction of O^6^-methylguanine-DNA methyltransferase promoter methylation	ResNet50, DenseNet201	Glioma patients	A pre-trained AI model was used in this study for the FE task.	T1-precontract, T1-postcontrast, T2-weighted and FLAIR MRI images	-	-	-	Both models had an accuracy of 100%
Li et al. (2023) [81]	Identification of 6-methyladenosis, 4-methylcytosine, and 5-hydroxymethylcytosine.	EpiTEAmDNA (a CNN-based model)	iDNA-MS, Hyb4mC, and DeepTorrent	AI was trained on the data methyl nucleotide data from all 15 species and then tested on a particular species dataset.	DNA sequences (*n* = 1,582,262) from 15 different species, including *Arabidopsis thaliana*, *Caenorhabditis elegans*, *Drosophila melanogaster*, *Escherichia coli*, and *Homo sapiens*	-	-	-	Average accuracy in 29 datasets: 88.62%
Salvatore et al. (2023) [82]	Identification of DNA regulatory elements	ChromTransfer	ENCODE project, JASPAR 2022 motif database	A pre-trained model on ENCODE data to identify regulatory element activities of DNA sequences	DNA sequences of six cell lines A549	0.86	0.42	F1-score for: A549: 0.86	-
					HCT116	0.79	0.4	HCT116: 0.8	-
					HepG2	0.89	0.74	HepG2: 0.79	-
					GM12878	0.85	0.49	GM12878: 0.8	-
					K562	0.87	0.45	K562: 0.86	-
					MCF7	0.85	0.64	MCF7: 0.73	-
Mehmood et al. (2024) [83]	Enhancer identification and their strength	A hybrid of ULMFIT, CNN, and attention layers	Benchmark dataset provided by Liu et al. [84] and independent data set.	AI was used to train and predict nucleotide sequences based on the previous sequence, and then the data were used to (1) classify encoder from non-encoder sequences and	DNA sequences	0.9097	0.9319	MCC: 0.686	Accuracy: 84.3%
				(2) predict the strength of the encoder		0.9902	0.988	0.774	87.5%
Yin et al. (2024) [85]	5-methylcytosine identification	NanoCon	Genome data of *A. thaliana* [86] *Oryza sativa* [87] NA12878 [88]	(1) A pre-trained model on *A. thaliana* was tested on *O. sativa*.	Nanopore sequencing data	0.9–1	approximately 0.9	-	Precision: 90–100%
				(2) A pre-trained model on *O. sativa* was tested on *A. thaliana*		0.9–1	0.7–0.8	-	40–50%
				(3) Training on CHG motifs and predicting 5-methylcytosine in CpG		0.5105	0.9344	F1 score: 0.8393	-
Yao et al. (2024) [89]	4-methylcytosine identification	DeepSF-4mC	The dataset provided by Zeng et al. (2020) [90]	They trained a CNN model on the DNA sequence of all three species and tested it on each species.	DNA sequences from *A. thaliana*	-	-	0.863/0.722	Accuracy: 86.1%
					*C. elegans*	-	-	0.855/0.814	90.7%
					*D. melanogaster*	-	-	0.888/0.772	88.5%
Zhang et al. (2024) [91]	eGene identification	TLegene	TCGA, GTEx project, Geuvadis projects	The model trained on the GTEx projects was tested on TCGA (cancers) and Geuvadis projects (non-cancer). Their four models identified between 310–325 significant genes.	Cis-single nucleotide polymorphism data	-	-	-	-

* Some studies applied TL in different ways and reported their results. In these cases, we reported the results of each approach separately by numbering them. AI: artificial intelligence, biLSTM: bidirectional long short-term memory, AUPRC: area under the precision–recall curve, AUROC: area under the receiver operator curve, CNN: convolutional neural network, FE: feature extraction, TF: transcription factor, FLAIR: fluid attenuated inversion recovery, MCC: Matthews correlation coefficient, MRI: magnetic resonance imaging, RNN: recurrent neural networks, TL: transfer learning.

## Data Availability

We have included all relevant information in this article; if further clarification is required, please get in touch with the corresponding author.
